# Limited Effects of Temperature on Captive Eastern Hellbender (*Cryptobranchus alleganiensis alleganiensis*) Growth

**DOI:** 10.1002/zoo.70086

**Published:** 2026-06-15

**Authors:** Kylie S. Soniak, Rod N. Williams, Lewis Single, Nicholas G. Burgmeier, Jason T. Hoverman

**Affiliations:** ^1^ Department of Forestry and Natural Resources Purdue University West Lafayette IN USA; ^2^ Office of the Provost Texas Tech University Lubbock TX USA; ^3^ Indianapolis Zoo Indianapolis IN USA

**Keywords:** captive propagation, developmental plasticity, husbandry, juvenile development, thermal effects

## Abstract

Amphibians are among the most threatened vertebrates, with 41% of species at risk of extinction due to factors such as habitat loss, pollution, and climate change. One such species, the Eastern Hellbender (*Cryptobranchus alleganiensis alleganiensis*), has experienced significant population declines, particularly in Indiana. Conservation efforts, including captive breeding and rearing, have been initiated to mitigate these losses. However, the rearing conditions that optimize the growth of this species in captivity are understudied. Higher temperatures are known to increase amphibian growth, but many captive rearing facilities rear Eastern Hellbenders at relatively low temperatures (e.g., 16°C). Our goal was to assess the effects of rearing temperature on Eastern Hellbender growth under captive rearing conditions. Eastern Hellbenders (*N* = 207) were reared at two temperatures (16°C and 21°C) at the Indianapolis Zoo. Every 8 weeks from February 2023 to July 2024, we collected mass and total body length on each individual to assess changes over time. Contrary to predictions, we found no evidence that higher temperatures increased the growth (mass or total length) of Eastern Hellbenders. This suggests that Eastern Hellbender growth is unlikely to be influenced by the rearing temperatures used at most captive facilities. These results challenge assumptions about the role of temperature in captive rearing outcomes and suggest that future research focus on other variables (e.g., diet, density) that might enhance captive‐rearing efficiency.

## Summary

- For captive‐reared Eastern Hellbenders, there was no influence of rearing temperature (16°C *vs.* 21°C) on growth.
- Our results suggest that Eastern Hellbender captive rearing programs seeking to optimize growth focus on other variables (e.g., density, diet) to improve the success of reintroductions.

## Graphical abstract

While higher temperatures are generally expected to increase amphibian growth, our experiments with captive‐reared Eastern Hellbenders found no evidence that growth increased at a rearing temperature of 21°C relative to 16°C. As Eastern Hellbender reintroduction efforts move forward, a focus on other conditions, such as density or diet, might be more valuable for optimizing captive rearing practices.

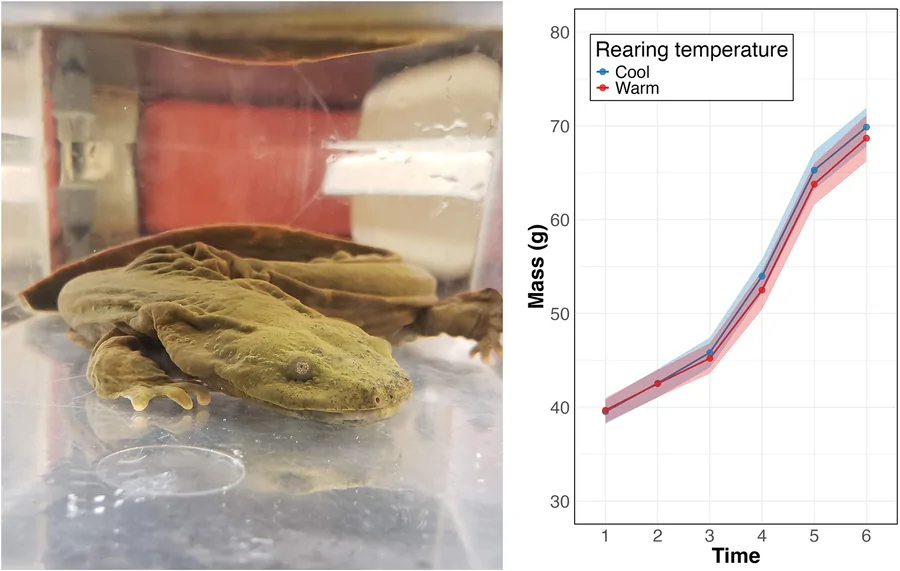

## 1 Introduction

Amphibians make up the largest group of endangered species with 41% of species listed as threatened (Luedtke et al. 2023). Consequently, conservation efforts have been initiated globally to minimize the loss of amphibian species. These endeavors encompass a range of conservation methods, including habitat restoration, governmental protection of crucial ecosystems, and captive breeding and propagation programs (Griffiths and Pavajeau 2008). Captive rearing, in particular, has become a powerful management tool for endangered species conservation programs. While captive rearing has been used effectively for a small subset of species, it remains novel for others, like the Eastern Hellbender (*Cryptobranchus alleganiensis alleganiensis*).

The Eastern Hellbender is a fully aquatic salamander that inhabits streams and rivers. It has faced precipitous population declines due to habitat degradation, water pollution, and disease (Briggler et al. 2007; Wheeler et al. 2003). Populations have experienced the steepest declines in the upper Midwest, including Indiana (Burgmeier et al. 2011b; Olson et al. 2013). Previous conservation efforts in Indiana have focused on captive propagation and conditioning, with the main objectives to increase population size and post‐release survival (Kenison et al. 2018; Kenison and Williams 2018a, 2018b). One potentially key component of increasing post‐release survival in Eastern Hellbenders is increasing growth rates to produce large individuals at a faster rate for release. Previous research with captive fish has shown that increasing growth rates improves success upon release because larger individuals often have enhanced physical strength and endurance, allowing them to avoid predators and cope with environmental stressors (Fraser 2008). Thus, research that examines how captive husbandry conditions and techniques affect growth rates can play a critical role in conservation efforts.

As ectotherms, amphibians are sensitive to temperature (Taylor et al. 2021). Between the critical thermal minimum and maximum, most functions (e.g., activity, physiology, feeding) of amphibians generally increase with temperature (Taylor et al. 2021). This has frequently been observed with amphibian growth rates (Berven 1982; Bonino et al. 2020; Smith‐Gill and Berven 1979). Given that one of the goals of captive rearing programs is to produce large individuals for reintroduction efforts, increases in rearing temperature could help facilitate growth and the pace of conservation efforts. To date, the influence of temperature on Eastern Hellbender growth has been unexplored. Most captive husbandry programs rear Eastern Hellbenders between 14°C and 18°C, depending on the season. However, Eastern Hellbenders in natural streams can experience temperatures up to 26°C in the summer (Burgmeier et al. 2011a). If Eastern Hellbender growth is responsive to increased temperatures, captive husbandry approaches could be refined to improve conservation efforts. To examine this question, we conducted a controlled laboratory experiment manipulating rearing temperature (16°C or 21°C) to test the hypothesis that Eastern Hellbender growth increases with temperature.

## 2 Materials and Methods

### 2.1 Hellbender Collection and Husbandry

The study was conducted at the Indianapolis Zoo under laboratory conditions. Eastern Hellbender eggs (*n* = 334) were collected in September 2020 from a creek in Kentucky, which remains unnamed to protect the sensitive population. The eggs were collected from a single nest rock with a single guarding male. We did not conduct genetic testing to determine if the eggs were from a single female or multiple. However, given the size of the nest, we suspect they were from a single female. Immediately after collection, the eggs were transported to the Indianapolis Zoo in an aerated cooler to ensure their viability. Upon arrival, they were placed in a tank equipped with a modified Kreisel system, providing optimal conditions for development until hatching (Greve 1968). Upon hatching, individuals were maintained under laboratory conditions until the start of the experiment in 2023.

### 2.2 Experimental Design

For the experiment, Eastern Hellbenders were housed in two experimental systems, each containing two raceways. System 1 maintained a constant water temperature of 16°C (cool) while System 2 maintained a water temperature of 21°C (warm). We selected 16°C to represent conditions typical of a husbandry facility and 21°C to represent a temperature within the range found during the summer in Indiana streams (Burgmeier et al. 2011a). Temperatures were monitored continuously with water‐resistant thermometers. The dimensions of all tanks were 2.4 m long, 0.6 m wide, and 0.6 m high. We split 207 individuals between the two systems, with system 1 receiving 104 individuals (52 per tank) and system 2 receiving 103 individuals (52 and 51 individuals per tank). The raceways contained a mix of stones, terracotta roof tiles, PVC tubes, and ceramic floor tiles for hides. Temperature and density conditions were maintained for all individuals from February 2023 until the end of the experiment in July 2024. We measured mass (g) and total length (cm) for each individual every 8 weeks. We used spot pattern mapping (see below) for individual identification.

### 2.3 Spot Pattern Mapping

We used a systematic approach to ensure accurate identification of individual Eastern Hellbenders (Kenison and Williams 2018a). Individuals were placed in a narrow container to limit excessive movement and were oriented as straight as possible for precise imaging. We situated an Olympus Tough TG‐6 camera 20.32 cm directly above the individual and photographed the dorsum and tail regions. During photography, we noted any distinctive spots or physical attributes that could aid in identification. After the image was taken, we relocated the individual to a holding tank before returning it to the experimental tank. We repeated this procedure systematically until all individuals within the tank had been documented.

### 2.4 Feeding

During the experiment, Eastern Hellbenders were fed an 11% food ration based on their body mass. We used measurements of mass from our data collection samples conducted every 8 weeks to adjust the feeding ration during the experiment. Eastern Hellbenders were fed frozen bloodworms and/or mysis shrimp every other day.

### 2.5 Statistical Analyses

The response variables in our analyses were Eastern Hellbender mass and total length over time (i.e., growth). Both response variables were log‐transformed for analysis. Our explanatory variable was temperature. Because we were able to track individuals, we used linear mixed‐effects models to assess the effects of temperature on growth across the data collection periods. In our models, we included tank and individual as random effects. There were mild violations (e.g., heteroscedasticity) of model assumptions in the dataset. However, mixed models are relatively robust to some heteroscedasticity (Schielzeth et al. 2020). Analyses were conducted using R Statistical Software v4.4.2 (R Development Core Team 2024) with the packages lme4, lmerTest, car, and DHARMa (Bates et al. 2015; Fox and Weisberg 2019; Hartig 2026; Kuznetsova et al. 2017).

## 3 Results

For both mass and total length, we found that temperature did not influence growth (Table 1; Figure 1). Mass and total length increased over time, but there was no influence of temperature or interactive effects between temperature and sample collection date. At the conclusion of the study, the mean ± 1 SD for mass was 81.19 ± 16.28 g, and total length was 26.92 ± 1.72 cm. Overall, mass increased by 113% and total length by 33% during the experiment. The full dataset is available in the Supporting Information.

**Table 1:** Results of linear mixed‐effects models examining the effects of temperature, sample collection date, and their interaction on Eastern Hellbender mass and total length. Bold *p*‐values are significant (*p* ≤ 0.05).

Factor	Mass			Total length		
	*X* ^*2*^	*df*	*p* value	*X* ^*2*^	*df*	*p* value
Temperature	0.20	1	0.657	1.53	1	0.215
Sample collection	3915.54	5	**< 0.001**	3388.90	5	**< 0.001**
Interaction	2.13	5	0.831	4.33	5	0.503

**Figure 1:**
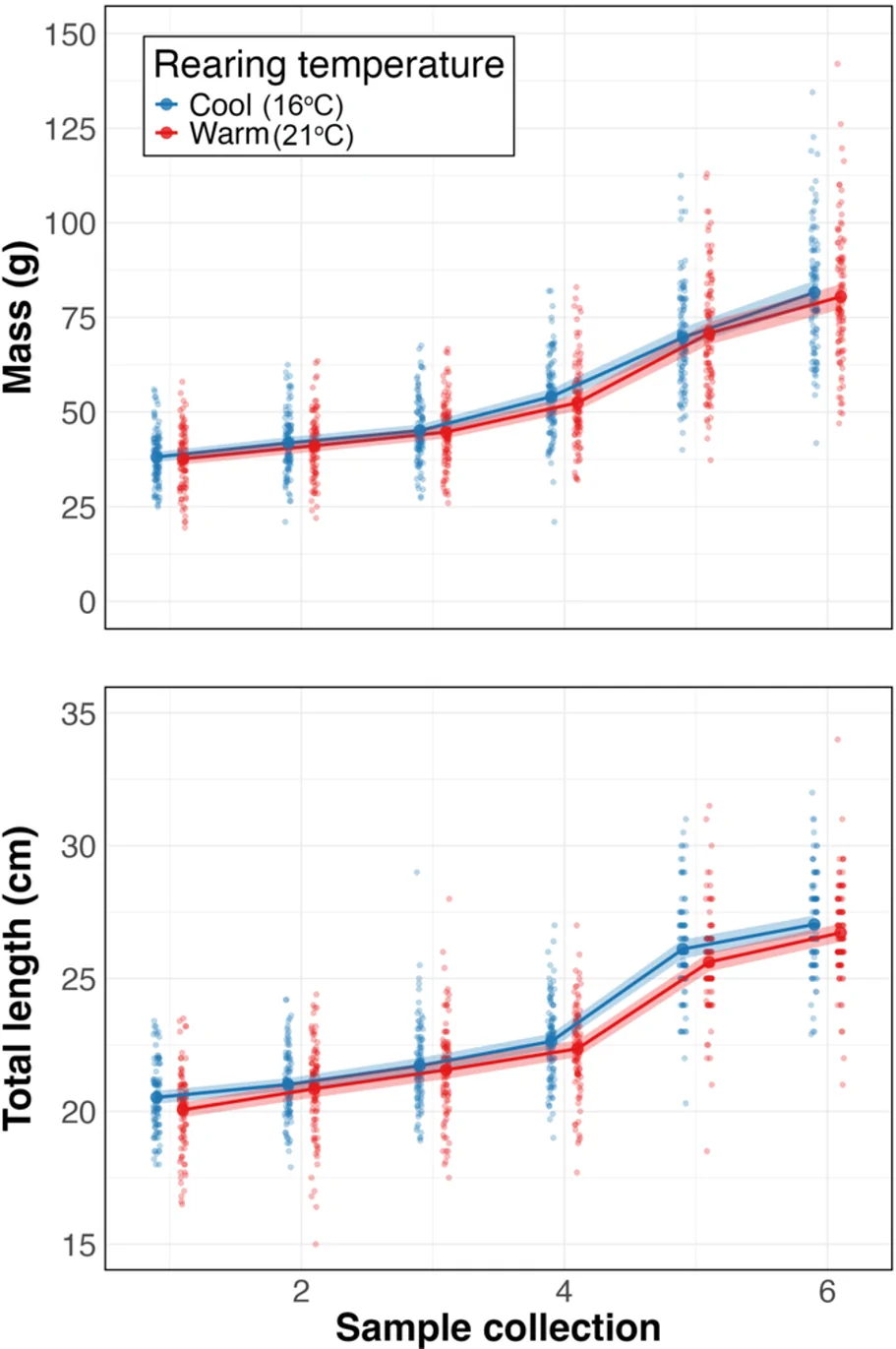
Plots of Eastern Hellbender mass and total length over time for each temperature treatment (Cool = 16°C, Warm = 21°C). The sample collection dates were 8 weeks apart during the experiment. The shaded region around each temperature treatment line is the 95% confidence interval and each point represents an individual.

## 4 Discussion

As ectothermic organisms, amphibians rely on external temperatures to regulate their body functions. As such, temperature plays a crucial role in the growth and development of amphibians, influencing their metabolic rates, physiological processes, and overall survival (Taylor et al. 2021). However, counter to our predictions, we found no evidence of increased Eastern Hellbender growth at a temperature of 21°C (warm) compared to 16°C (cool). It is important to note that mass increased by 113% and total length increased by 33% over the experiment, suggesting that there was capacity for growth changes associated with our treatments. Nonetheless, the lack of significant effects in our study could have been driven by our cohort of individuals being 2 years old. It is possible that individuals at this age are less responsive to environmental changes. For instance, previous laboratory studies with embryos and early‐stage larvae of Japanese Black Salamanders (*Hynobius nigrescens*) and Rough‐skinned Newts (*Taricha granulosa*) found that warmer temperatures (20°C and 21°C, respectively) increased growth rates relative to cooler temperatures (15°C and 14°C, respectively) (Morii et al. 2024; Smith et al. 2015). Based on this research, future studies conducted with younger Eastern Hellbender age classes could provide more definitive data on growth patterns in response to temperature.

Another possible reason for the lack of temperature effects could be related to the relatively small 5°C difference between our treatments. While our choice of treatments reflected typical differences in captive rearing facilities, the differences may not have been large enough to produce significant variation in growth. Given that some Eastern Hellbender populations can encounter temperatures above 21°C in the field during the summer (Burgmeier et al. 2011a), it would be valuable to explore a broader temperature gradient in laboratory experiments to determine growth responses under field conditions and assess whether higher temperatures in captivity will increase Eastern Hellbender growth.

In amphibians, developmental rates are generally more sensitive to temperature than growth rates. For example, at colder temperatures, developmental rates of larval amphibians are reduced while growth continues, albeit at slower rates (Riha and Berven 1991; Ruthsatz et al. 2018). At higher temperatures, amphibians often display shorter times to maturity and first reproduction (Tilley and Bernardo 1993). Our study was not long enough to explore potential reproductive responses. However, it would be interesting to assess whether time to maturity could be influenced by temperature in future long‐term experiments. If Eastern Hellbenders can reach maturity sooner under warmer rearing conditions, reintroduction programs may have greater success because individuals could potentially reproduce sooner after release.

While more research is needed to assess the influences of temperature on Eastern Hellbenders, efforts might be better spent on other factors that influence growth in captivity. For instance, there has been limited work on the optimal densities and feeding rations for Eastern Hellbenders in captivity. Because a major goal of captive‐rearing programs is to provide enough food and space to produce large, healthy individuals, experimental approaches that generate reliable data on these key factors would be invaluable. In conclusion, while this study has provided a foundation, continued research will be crucial for optimizing captive rearing practices and improving the long‐term conservation of this threatened species.

## Author Contributions

**Kylie S. Soniak:** conceptualization, data curation, formal analysis, investigation, methodology, visualization, writing – original draft. **Rod N. Williams:** conceptualization, data curation, funding acquisition, investigation, methodology, project administration, resources, supervision, writing – review and editing. **Lewis Single:** conceptualization, funding acquisition, investigation, methodology, project administration, resources, supervision, writing – review and editing. **Nicholas G. Burgmeier:** conceptualization, funding acquisition, methodology, writing – review and editing. **Jason T. Hoverman:** data curation, formal analysis, methodology, visualization, writing – review and editing.

## Ethics Statement

All work was in accordance with protocols approved by the Purdue Animal Care and Use Committee (1406001094) and the Indiana Department of Natural Resources (Indiana Scientific Purposes License 2669).

## Conflicts of Interest

The authors declare no conflicts of interest.

## Supporting information

Supporting File

## Data Availability

The data that supports the findings of this study are available in the Supporting Information of this article. All associated data presented in this article are available within the Supporting Information.
